# Supplementary material for: Persistence of metric biases in body representation during the body ownership illusion
Source: PLoS One. 2022 Jul 26;17(7):e0272084. doi: 10.1371/journal.pone.0272084 (PMC9321438; doi:10.1371/journal.pone.0272084)
Supplement: Attachment — Submitted filename: Response_to_Reviewers.docx [file pone.0272084.s002.docx]

**Response to Reviewers**

**Reviewer #2**

- My one remaining issue is that it should be clear from the abstract that the main measure in this study was a questionnaire.

Thank you for your comment. We added the lines to the abstract about the usage of a questionnaire as the main measurement method in the study (line 37-38).
